# Reorienting the Fab Domains of Trastuzumab Results in Potent HER2 Activators

**DOI:** 10.1371/journal.pone.0051817

**Published:** 2012-12-20

**Authors:** Justin M. Scheer, Wendy Sandoval, J. Michael Elliott, Lily Shao, Elizabeth Luis, Sock-Cheng Lewin-Koh, Gabriele Schaefer, Richard Vandlen

**Affiliations:** 1 Department of Protein Chemistry, Genentech Inc., South San Francisco, California, United States of America; 2 Department of Research Oncology, Genentech Inc., South San Francisco, California, United States of America; 3 Department of Biostatistics, Genentech Inc., South San Francisco, California, United States of America; Bioinformatics Institute, Singapore

## Abstract

The structure of the Fab region of antibodies is critical to their function. By introducing single cysteine substitutions into various positions of the heavy and light chains of the Fab region of trastuzumab, a potent antagonist of HER2, and using thiol chemistry to link the different Fabs together, we produced a variety of monospecific F(ab′)_2_-like molecules with activities spanning from activation to inhibition of breast tumor cell growth. These isomers (or bis-Fabs) of trastuzumab, with varying relative spatial arrangements between the Fv-regions, were able to either promote or inhibit cell-signaling activities through the PI3K/AKT and MAPK pathways. A quantitative phosphorylation mapping of HER2 indicated that the agonistic isomers produced a distinct phosphorylation pattern associated with activation. This study suggests that antibody geometric isomers, found both in nature and during synthetic antibody development, can have profoundly different biological activities independent of their affinities for their target molecules.

## Introduction

Recently it has been shown that cysteines introduced into various places on monoclonal antibodies allow for the production of site-specific, homogeneous drug-antibody conjugates with many useful properties [1], [2], [3]. The introduced cysteines are generally outside of the complementarity-determining regions (CDRs) of the antibodies so as to not perturb antigen recognition. Multiple sites on the antibody framework have been evaluated as to ease of reaction with maleimide-containing molecules, the homogeneity of the resultant conjugates and their stability in vitro and in vivo after conjugations [4]. In general, this approach has generated antibody variants with multiple desirable attributes.

We used this paradigm to develop a combinatorial platform for producing novel multispecific and monospecific antibody variants. From a combinatorial chemistry point of view, the availability of specific, highly reactive sites at various places on the Fab domain of antibodies should allow for facile generation of Fab′2-like molecules containing the Fabs from two different antibodies (e.g. bispecific molecules) or containing two different cysteine mutations from the same parent antibody (e.g. monospecific molecules). In both cases one has exquisite control of the orientation or geometry between the two Fab arms as defined by the location of the cysteine mutations on each Fab. In addition, control over the flexibility between the Fab arms of the molecule and the distance between the antigen binding sites is possible. We reasoned that an approach that used engineering and chemical linking could provide a method for designing and developing molecules with novel activities. These are capabilities that are not available with a typical antibody format, but are more similar to domain fusions such as diabodies, where geometry and linker design are important for producing a stable, functional molecule. In this report we describe our bis-Fab technology and its application to the generation and modulation of activity of bispecific and monospecific Fab′2-like molecules directed against receptors of the epidermal growth factor family.

Epidermal growth factor receptor (EGFR, ErbB, or HER) family members are essential regulators of cell growth, development and normal adult cellular physiology and are implicated in many abnormal physiological conditions such as cancers [5], [6]. Attempts to inhibit the activity of these receptors have resulted in potent antibody pharmaceuticals that are effective in treating cancer. Examples of therapeutic antibodies include trastuzumab (Herceptin®), pertuzumab, cetuximab, and panitumumab. These antibodies bind to different HER-family members (trastuzumab and pertuzumab bind HER2; cetuximab and panitumumab bind EGFR) and different domains within the same protein target (trastuzumab, domain IV; pertuzumab, domain II; cetuximab and panitumumab, domain III). These antibodies are used in a variety of clinical applications; for example, trastuzumab is highly effective in treating certain forms of breast cancer. There are limitations to its effectiveness, however, in part because of heterodimerization among EGFR family members and the involvement of other growth factor receptors [7]. Therefore, we developed a Fab recombination approach to identify Fab′2-like molecules that would more effectively inhibit these targets.

During our studies we created new antibody variants targeting HER2 that are identical in the complementarity-determining regions, and but vary greatly in the relative geometry of the Fab domains. These antibody variants are monospecific for HER2 yet show a spectrum of biological activities that are different from the parent antibody from which they were derived. We have used these unique HER2 selective probes as tools to detail the interaction of trastuzumab with HER2 on breast cancer cells. As an approach to antibody design, this technique shows promise for not only identifying tools to probe signal transduction pathways, but also to meet emerging engineering challenges of developing more effective and specific antibody-based therapeutics.

## Experimental Methods

### Bis-Fab Synthesis

**Figure 1:**
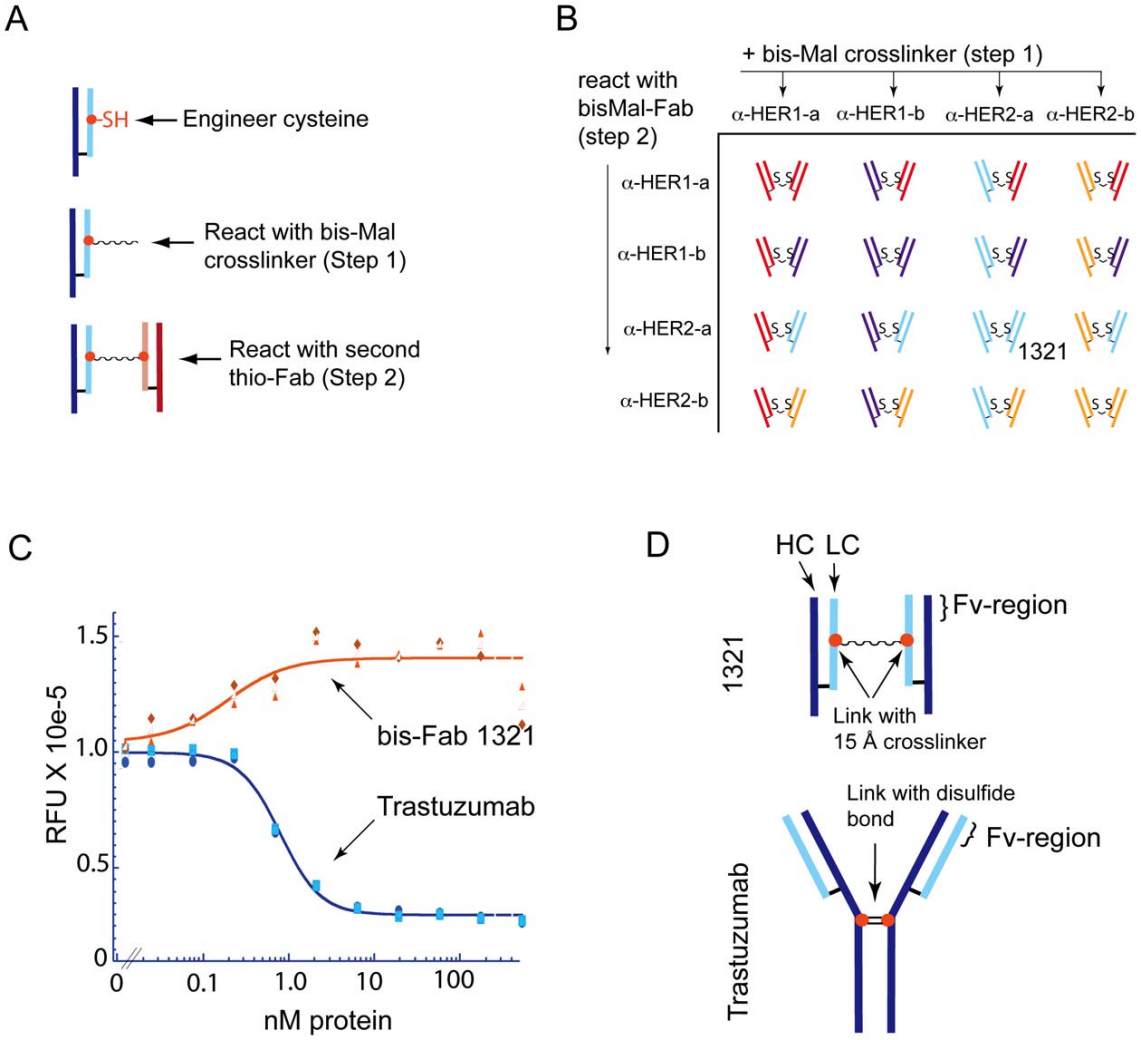
Agonist activity of a trastuzumab isomer. (a) A brief description of the bis-Fab synthesis process is illustrated here. The thio-Fabs of interest are produced by engineering an unpaired cysteine into the Fab region of an antibody followed by recombinant expression and isolation of the thio-Fab containing the unpaired cysteine. A homobifunctional crosslinking reagent is then used in a two-step process to efficiently couple two thio-Fabs together. A more detailed explanation of the procedure can be found in the Experimental Methods and **Figure S1**. (b) A matrix combination of thioFabs using the two-step synthesis process was used to produce a panel of bis-Fab molecules for use in biological and biochemical assays. Two Fabs targeting EGFR (α-HER1-a targets domain III and α-HER1-b targets domain III) and two Fabs targeting HER2 (α-HER2-a derived from trastuzumab targets domain IV and α-HER2-b derived from pertuzumab targets domain II) were used to construct the matrix. (c) Trastuzumab and bis-Fab 1321, consisting of two trastuzumab Fabs linked together at position 110 in the light chain, were examined for their effect on cell growth. Increasing concentrations of trastuzumab (blue line) or bis-Fab 1321 (orange line) were added to BT474 cells and cell proliferation was measured after five days using AlamarBlue staining. The relative fluorescence units are reported for the different treatment concentrations. Individual data points for two independent experiments are shown in the plot as well as an average of the two, which are represented by the lines and open shapes. Trastuzumab analog (bis-Fab 1321) showed agonistic activity as measured by increased cell proliferation, whereas trastuzumab inhibited cell proliferation. (d) The schematic illustrates the site of covalent attachment between the Fabs of the parent antibody trastuzumab and bis-Fab 1321. Although the global conformations of the Fab domains are unknown, this figure highlights the distinct difference in the points of connection. The native interchain disulfides (hinge region, near HC-228) in the heavy chains of trastuzumab provided the covalent attachment site for the Fab arms in the antibody. In contrast, bis-Fab 1321 was covalently linked through light chains at LC-110 using a bis-maleimido crosslinker. The resultant molecules presented Fab Fv-regions in different relative orientations.

Thio-Fabs with an unpaired cysteine were derived from various sources as described in the following sections. After isolation and clean-up of the thio-Fabs such that the thiol from the unpaired cysteine was presented, the first thio-Fab was transferred into a buffer containing 25 mM MES, pH 5.8, 2 mM EDTA, and 300 mM NaCl (**Figure S1**, panels 1–3). To this, a five-fold molar excess of crosslinker bis(maleimide) amine TFA (Quanta Biodesign) was added to the thio-Fab (1 mg/mL) with mixing (**Figure 1**, panel 4). After four hours the reaction was complete and the mixture was concentrated to a volume suitable for gel filtration on a 22 mL S-200 Tricorn column (GE Healthcare). The isolated thio-Fab plus crosslinker species was then added to the second thio-Fab in the same buffer and concentrated to 5 mg/mL or greater to drive the reaction to completion (2 hrs) (**Figure S1**, panel 5). The completed reaction was purified by gel filtration and the dimeric peak was collected (**Figure S1**, panel 6). The reaction progress during both steps was monitored by mass spectrometry and clearly showed the presence of both reactants and the formation of the bis-Fab product (**Figure S1**, panel 5). The purity of the desired product after the second gel filtration was determined by mass spectrometry and SDS-PAGE. Upon reduction and SDS-PAGE analysis, irreversible crosslinking was observed by the presence of a 50 kD band representing non-reducible crosslinked chains (**Figure S1**, panel 6).

### Thio-Fab expression and purification in E. coli

Protein expression in *E. coli* was carried out either by overnight culturing in shake flasks or in a 10-liter fermentor as described previously [8], [9]. *E. coli* cell pellets expressing recombinant thio-Fabs or recombinant hinge-cys-Fabs were resuspended in a buffer containing 25 mM Tris, pH 7.5, 125 mM NaCl, 5 mM EDTA (TEB) and lysed using a microfluidizer. The extract was treated with the flocculent polyethyleneimine (0.4%) adjusted to pH 9.0 for 1 hour with stirring followed by centrifugation for 45 minutes at 15,000×g. Thio-Fabs or hinge-cys-Fabs were purified by standard procedures using Protein G and cation exchange chromatography. Specifically, the supernatants were filtered through a 0.22 micron filter and then directly applied to a Hi-Trap Protein G resin (GE Healthcare). Elution of the Fab was achieved with 0.2 M acetic acid followed by capture on an SP-HP cation exchange column (GE Healthcare). Thio-Fabs or hinge-cys-Fabs were eluted with a 10 CV gradient of 0–1 M NaCl. Purified thio-Fabs were characterized by SDS-PAGE and mass spectrometry. These characterizations often showed mass increases of 275 Da and 306 Da corresponding to disulfide adducts on the unpaired cysteine. These adducts were removed by reduction and oxidation to prepare the thio-Fabs for crosslinking with bis-maleimide (**Figure S1**, panel 1–3). Thio-Fabs were reduced for 24 hrs by the addition of 2 mM tris(2-carboxyethyl) phosphine HCl (TCEP) (Thermo Fisher Scientific) in a buffer containing 25 mM MES, pH 5.8, 300 mL NaCl, and 5 mM EDTA and re-oxidized by the addition of 5 mM dehydroascorbic acid (DHAA) (Sigma-Aldrich). The isolated thio-Fabs were analyzed by SDS-PAGE and mass spectrometry to ensure that the proteins were properly reduced and oxidized.

### Additional methods for preparation of thio-Fabs and hinge-cys-Fabs

Several additional approaches were used to create antibody fragments with sulfhydryl groups (**Figure S2A**). In one approach, cysteine substitutions were introduced into antibody constructs at various positions in the constant domains of light chains or heavy chains by site-directed mutagenesis to create thio-Mabs as described previously [4]. Thio-Fabs were generated enzymatically from thio-Mabs (CHO thio-mAb) by diluting thio-Mabs to 1 mg/mL in 25 mM Tris, pH 8.0, and enzymatically digesting at 37°C for 1 hr using Lys-C (Wako Chemicals USA, Inc.) at a 1∶1000 (wt∶wt) ratio of enzyme to antibody. The Lys-C digestion was stopped with 5 µM of the protease inhibitor tosyl-L-lysine chloromethyl ketone (TLCK) (Bachem) and purified by cation ion exchange chromatography on a 5 mL Hi-Trap SP FF column (GE Healthcare) using a 50 mM sodium acetate buffer and a 0–300 mM NaCl 10 CV gradient. Another approach was used for antibodies lacking an engineered cys residue and relied upon the native cys residue(s) present in the hinge region of IgG1. This method was used to produce “hinge-cys-Fabs” (HC-228) and is described in further detail below.

For the preparation of hinge-cys-Fabs from native antibodies that did not contain an engineered cysteine for use in synthesis reactions, we used the following procedure. Trastuzumab was digested with pepsin (1% w/w) by treatment in sodium acetate buffer at pH 4.5. After digestion for 1 hour, the F(ab′)_2_ was isolated from the digestion mixture by capture on an SP-HP cation exchange resin and purified by a 10 CV salt gradient of 0–1 M NaCl. The F(ab′)_2_ was then reduced with 1 mM TCEP in a buffer containing 25 mm MES, pH 5.8, 2 mM EDTA, and 300 mM NaCl and the Fabs were oxidized by the addition of 5 mM dehydroacorbic acid (DHAA) to reform the disulfide bond between the heavy chain and light chain. We routinely observed that under these reaction conditions, only the disulfide between the heavy chain and light chain was reformed; the two cysteine residues in the hinge region remained reduced.

The two thiols (cys residues) at the hinge were then reacted with 1 equivalent of N-ethylmaleimide (NEM) (Sigma Aldrich). The resultant mixture containing singly-modified, doubly modified and unmodified Fabs were then reacted with an excess of the bis-maleimido crosslinker (**Figure S2B**). This reaction yielded three products: Fabs with one crosslinker and one NEM, Fabs with two NEM, and Fabs containing only one crosslinker (**Figure S2B**). The Fabs containing only one crosslinker (no NEM) were found to have no free cysteine. Thus, under these reaction conditions, a single crosslinker reacted very efficiently with both cysteines resulting in a molecule where the cysteines have been cyclized by the crosslinker. The material comprising the above three reaction products was purified from the reaction mixture (to remove unwanted reaction components) by gel filtration and used in coupling to other thio-Fabs. Only hinge-cys-Fabs containing one crosslinker and one free-maleimido were able to react in the bis-Fab synthesis reactions described in the beginning section.

### Cell-surface affinity and hydrodynamic radius

Cell-surface binding constants were determined in BT474 cells by Scatchard analysis using ^125^I-labeled antibodies or bis-Fabs. Data reported in **Table 1** are representative of two independent experiments.

**Table 1:** Cell-surface binding and solution state properties of HER2 agonists and antagonists.

Molecule†	Activity Type‡	Cell-surface *K_D_* (nM)	Sites Per Cell (×10^−5^)	*R_hyd_*
1325 (HC118/LC205)	Agonist	1.9+/−0.3	3.2+/−0.2	3.70+/−0.11
1321 (LC110/LC110)	Agonist	2.5+/−0.6	2.8+/−0.1	3.66+/−0.11
1324 (LC205/LC205)	moderate antagonist	2.0+/−0.3	3.8+/−0.1	3.80+/−0.06
1329 (HC228/HC228)	Antagonist	1.3+/−1.3	n.d.	4.18+/−0.07
F(ab′)2	Antagonist	1.2+/−0.2	2.8+/−0.03	3.95+/−0.07
Trastuzumab	Antagonist	1.6+/−0.3	3.0+/−0.2	5.15+/−0.08
Fab trastuzumab	weak antagonist	5.7+/−1.2	5.5+/−0.3	2.46+/−0.11

† All molecules in this table are derived from trastuzumab. LC = light chain; HC = heavy chain. Numbers indicate the cysteine substitution position.

‡ Activity was determined by cell proliferation assays using BT474 cells.

***R_hyd_*** = hydrodynamic radius determined by size exclusion chromatography coupled-light scattering.

Standard deviation is represented by +/− values in the table.

Hydrodynamic radii (*R_hyd_*) were calculated using a Wyatt Technology Dawn Heleos II with QELS and Optilab Rex. Samples were run over a Shodex 803-kw column run in PBS with an additional 0.15 M NaCl to isolate a mono-dispersed peak. *R_hyd_* data points were chosen from 10 contiguous radii calculated at the peak maxima. Data reported in **Table 1** are the mean of 10 measurements including standard deviations from the mean. A Tukey test using GraphPad-Prism indicated all comparisons were statistically significant with a P value of <0.05 with the exception of bis-Fab 1329 compared to F(ab′)_2_.

### Model of bis-Fab-HER2 complexes

We generated models of how bis-Fabs may bind to the HER2 extracelluar domain (ECD) in a trimeric complex using simple manual methods and PyMol. One Herceptin Fab-HER2 ECD structure (PDB ID Code: 1N8Z) was used to generate a second structure. The two structures were maintained in the same plane in a similar manner to the cell membrane. The fourth domain of the ECD is the last domain before the transmembrane region. This was maintained as an inflexible plane. We then attempted to bring the structures together in different orientations assuming full rotational flexibility across the crosslinker. Two crosslinking sites were positioned near each other and a predetermined maximum distance was maintained. The distance between the crosslinked sites from the alpha-carbon in both of the cysteines used for crosslinking was measured using a thermodynamically minimized crosslinked structure using ChemDraw and was determined to be between 15–18 angstroms. The models show the only possible orientation that preserved the plane of the plasma membrane, maintained a crosslinking distance between 15–20 angstroms, and did not introduce steric clashes between the Fab domains for an antagonist or an agonist.

### Cell-proliferation assays

BT474 cells were purchased from American Type Cell Culture and maintained in RPMI medium supplemented with 10% fetal bovine serum. Cells (10,000/well) were plated in 96 well plates and treated the next day with the indicated concentrations of bis-Fabs or antibody in 1% serum-containing medium. After 5 days, AlamarBlue (Invitrogen) was added to the wells and fluorescence was read using a 96-well fluorometer with excitation set at 530 nm and emission at 590 nm. The results are expressed in relative fluorescence units (RFU), or as a percentage of growth compared to control group.

For the time course experiments, BT474 cells were grown in 50∶50 F12∶DMEM including 10% FBS, Glutamax (Gibco), 100 units/mL penicillin, and 100 ug/mL streptomycin. 15 cm tissue culture dishes were seeded with 1.5×10^6^ cells and grown for 24 hrs. Media was exchanged with 25 mL of fresh media containing 100 nM trastuzumab, 100 nM bis-Fab 1325, 10 nM heregulin, or no additional reagent (no treatment). Cells were harvested with 0.05% Trypsin every 12 hours for each treatment group and cells were counted with an Nexcelom Bioscience Cellometer Auto T4 cell counter. Each treatment group and time point was reported as an average from three independent plates.

### Pathway analysis by Western blot and ELISA

BT474 cells were seeded into 24 well plates at 5×10^5^ cells/well. The following day, cells were treated with trastuzumab, bis-Fab 1325, bis-Fab 1329, and control antibody (anti-gD) at 100 nM each for 10, 30 and 120 minutes at room temperature. The cells were lysed with nondenaturing lysis buffer containing 1% Triton (Cell Signaling Technology Cat#9803). Protein concentrations were determined and equal amounts of proteins were subjected to immunoblotting. The following antibodies were used for detection: pHER3 (#4791), pAKT (Ser473) (#9271), AKT (#9272), pMAPK (#9101), and MAPK (#9102) all from Cell Signaling Technology, HER3 (#SC-285, Santa Cruz Biotechnology), HER2 (#MS-730-P1, Neomarkers), pTyr (#525320, Calbiochem) and anti-tubulin (#T9026, Sigma).

The cell lysates described above were also evaluated with PathScan p-AKT1 (S473) Sandwich ELISA (Cat#7160, Cell Signaling Technology). Protein (10 µg) diluted in sample diluents was added to each well of the 96-well plate and the plate was incubated overnight at 4°C. After several wash steps, detection antibody was added and the plate was incubated for 1 hour at 37°C. Samples were washed 4 times, HRP-linked secondary antibody was added to the plate, and incubated for 30 minutes at 37°C. Plates were rinsed three times before the addition of substrate solution. After sufficient color development, 50 µL of 2.5 M H_2_SO_4_ was added to each well to terminate the reaction. Absorbance was measured at 490 nm with background subtracted at 650 nm. Samples were analyzed in triplicates and AKT phosphorylation levels of treated samples were compared to untreated control.

### Immunoisolation and quantitative mass spectrometry

A HER2-specific antibody (7C2, Genentech, Inc.) was used to isolate HER2 from BT474 cells either before or after the different treatments. The antibody-HER2 complex was captured from cell lysates using Protein A/G resin (GE Healthcare) in the presence of phosphatase and protease inhibitors. Eluted proteins from immuno-captures were prepared for mass spectrometry by reduction in SDS-sample buffer containing 50 mM DTT (Pierce Biotechnology) at 90°C for 5 min followed by alkylation with 0.176 M n-isopropyl iodoacetamide (synthesized in house) at room temperature for 20 min. Samples were separated on a 4–20% SDS-PAGE gel (Invitrogen) (200 V, 50 Min, 5 W) and the gel was fixed in 10% acetic acid/50% methanol (Burdick and Jackson) for 30 min. The fixed gel was stained overnight in Coomassie Brilliant Blue stain, destained and gel bands around 175 kDa (HER2 migration region) were excised, washed in 50 mM ammonium bicarbonate (Sigma) containing 5% acetonitrile (Burdick and Jackson, Muskegon, MI) (100 µL, 20 min) and followed by 50 mM ammonium bicarbonate in 50∶50 acetonitrile: water (100 µL, 20 min). Gel pieces were dehydrated with acetonitrile and digested with trypsin (Promega), in ammonium bicarbonate pH 8, 0.2 µg overnight at 37°C. Digestions of chymotrypsin or endoproteinase Glu-C (Roche) were also performed to obtain near complete coverage of the protein.

Peptides were extracted from the gel slices in 50 µl of 50∶50 v/v acetonitrile: 1% formic acid (Sigma) for 30 min followed by 50 µl of pure acetonitrile. Extractions were pooled and evaporated to near dryness and were reconstituted in 0.1% formic acid. Samples were injected *via* an auto-sampler onto a 75 µm×100 mm column (BEH, 1.7 micron, Waters Corp) at a flow rate of 1 µL/min using a NanoAcquity UPLC (Waters Corp). A gradient from 98% solvent A (water +0.1% formic acid) to 80% solvent B (acetonitrile +0.08% formic acid) was applied over 40 min. Samples were analyzed on-line *via* nanospray ionization into a hybrid LTQ-Orbitrap mass spectrometer (Thermo Fisher Scientific). Data was collected in data dependent mode with the parent ion being analyzed in the FTMS and the top 8 most abundant ions being selected for fragmentation and analysis in the LTQ. Tandem mass spectrometric data was analyzed using the Mascot search algorithm (Matrix Sciences, Boston, MA). Phosphorylation sites on HER2 were confirmed and localized by *de novo* interpretation. **Table S1** shows the complete phosphorylation coverage of HER2.

For sample preparation for quantitative mass spectrometry of the different phosphorylation sites, 15-cm tissue culture plates with 3.0×10^6^ BT474 cells per plate were exchanged into 25 mL of fresh media (as described above) and incubated at 37°C for 2 hours. At time zero, 100 nM trastuzumab, 100 nM bis-Fab 1325, 10 nM heregulin, or media was added to the cells. Treatment groups were incubated at 37°C for 10 minutes. After treatment, the media was replaced with 1 mL/plate of ice-cold lysis buffer (PBS, 10 mM NaFl, 1% CHAPS, 1% Triton X-100, phosSTOP phosphotase inhibitors (Roche) and cOmplete, EDTA-free protease inhibitors (Roche)). The cells were harvested using a cell lifter. Cells were further pulsed on ice briefly with a micro-sonication probe (low power for 1 second). Anti-HER2 antibody (22 µg) that binds domain I of HER2 (7C2, Genentech, Inc.) was added to the lysate and allowed to complex with HER2 in the presence of 100 uL Protein A/G Plus resin (Pierce Biotechnology) for 1 hour at 4°C with rotation. Lysate was removed by centrifugation and the resin was washed 3 times with ice cold PBS. Proteins were eluted from the capture beads by boiling in SDS-PAGE sample buffer and recovering the supernatant by filtration.

Isotopically-labeled synthetic peptides containing experimentally determined HER2 phosphorylation sites were custom made by Cell Signaling Technologies. A list of the phosphorylated peptides used and their non-phosphorylated analogs are provided in **Table S2**.

Dried gel extracted tryptic peptides were reconstituted in 0.1% formic acid containing 50 fmol/uL custom HER2 synthetic peptide mixtures for phosphorylation site quantification. Samples were injected onto the LTQ-Orbitrap and analyzed by Mascot. Phosphopeptide ions were compared to their synthetic analogs using the Quan Browser in Xcalibur (Thermo Scientific) and by manual peak area assignment. Three biological replicates of each immunoprecipitation of each treatment group were utilized for quantification and the resultant digests were analyzed in triplicate.

### Statistical methods

The variance component model was used to assess the sources of variability in the relative phosphorylation levels for each peptide within each group of treated samples. Total variation was apportioned among biological and technical replicates. Due to the nesting of injections within samples, the variance component model was also used to estimate the mean relative phosphorylation level for each peptide within each treatment group (**Table S1**) [10].

For each peptide, a mixed effects model was fitted to the relative phosphorylation level, with treatment as a fixed effect and sample as a random effect. Pairwise comparisons of groups were carried out using Tukey-Kramer's method to adjust for multiple comparisons. This controls the overall false positive rate associated with performing multiple statistical tests for each peptide. The analysis was conducted using the MIXED procedure in SAS version 9.1.3 [11]. Statistically significant changes with a p value<0.05 are shown.

## Results

### Bis-Fab approach for multi-targeting

Tumor cell growth is often driven by the activation of more than one signaling protein thus requiring multiple neutralization activities to provide effective therapies for certain cancers [12], [13]. One approach to build and design multi-targeted molecules is to use bispecific antibody technologies, whereby a single antibody-like molecule possesses two unique monoclonal activities [14], [15]. Because the most effective target combinations are not always known, we sought to enable rapid production and screening of combination molecules to identify those with desired efficacy. To do this we developed a synthetic process for rapidly combining Fabs using thiol chemistry targeting engineered cysteines as illustrated in **Figure 1A**. Our synthetic approach has hallmarks of being robust, rapid and general (described in more detail in **Figure S1**). We termed these antibody-like molecules bis-Fabs to include the bis-maleimido linker in the name and to distinguish them from a F(ab′)_2_. By using site-specific cysteine substitutions that have been previously characterized for efficient reactivity [4], quantitative conversion of a Fab containing a single unpaired cysteine (thio-Fab) to a Fab with a single bis-maleimide crosslinker has been obtained. The reaction product containing a single linker can then be reacted with the second thio-Fab and monitored by mass spectrometry for product appearance. Using two gel filtration steps during the synthesis we efficiently produced a single homogeneous species containing only the desired crosslinked molecule. This allows for recombination of virtually any Fab in a matrix format to synthesize a panel of related bivalent molecules derived from different antibodies (**Figure 1B**).

### Identification of a trastuzumab structural analog

As a proof of concept for our screening technology we targeted signaling receptors in the HER-axis because of their critical role in cancer and the availability of well-characterized antibodies [6], [16], [17], [18], [19], [20], [21]. Initially, we synthesized a matrix targeting HER2 and EGFR using four different thio-Fabs to identify the best combination of Fabs for designing a bispecific antibody. The four Fabs chosen for the matrix combinations were derived from trastuzumab (domain IV of HER2), pertuzumab (domain II of HER2), and two antibodies developed in-house targeting EGFR, anti-HER1-a (domain III) and anti-HER1-b (domain III). We observed a range of activities for the molecules, the most interesting of which was potent agonism of BT474 cell proliferation by a molecule composed of two identical trastuzumab Fabs (**Figure 1C**). This molecule, bis-Fab 1321, was covalently linked through a cysteine residue engineered at position 110 of the light chain. This linkage point was located in the loop (elbow region) connecting the variable domain and the constant domain. Coupling of two Fabs at this point resulted in a distinct rearrangement of their binding domains relative to each other as compared to the parent antibody (**Figure 1D**).

Although we suspected that different linkage positions could result in variable activity, it was remarkable that by simply changing the position of the covalent association between the Fabs we converted a potent antagonist into a potent agonist. This illustrated that a simple structural analog of an antibody was sufficient to impart radical changes in its biological activity. This observation is meaningful because, despite the importance of HER2 in driving cell proliferation, no ligand that directly binds HER2 to increase activity has been identified. Instead, HER2 activation occurs through dimerization with other ligand-binding family members [22]. Successful activation through heterodimerization is determined by the relative orientation of the receptor intracellular kinase domains and allosteric interactions.

### Trastuzumab linkage-matrix

**Figure 2:**
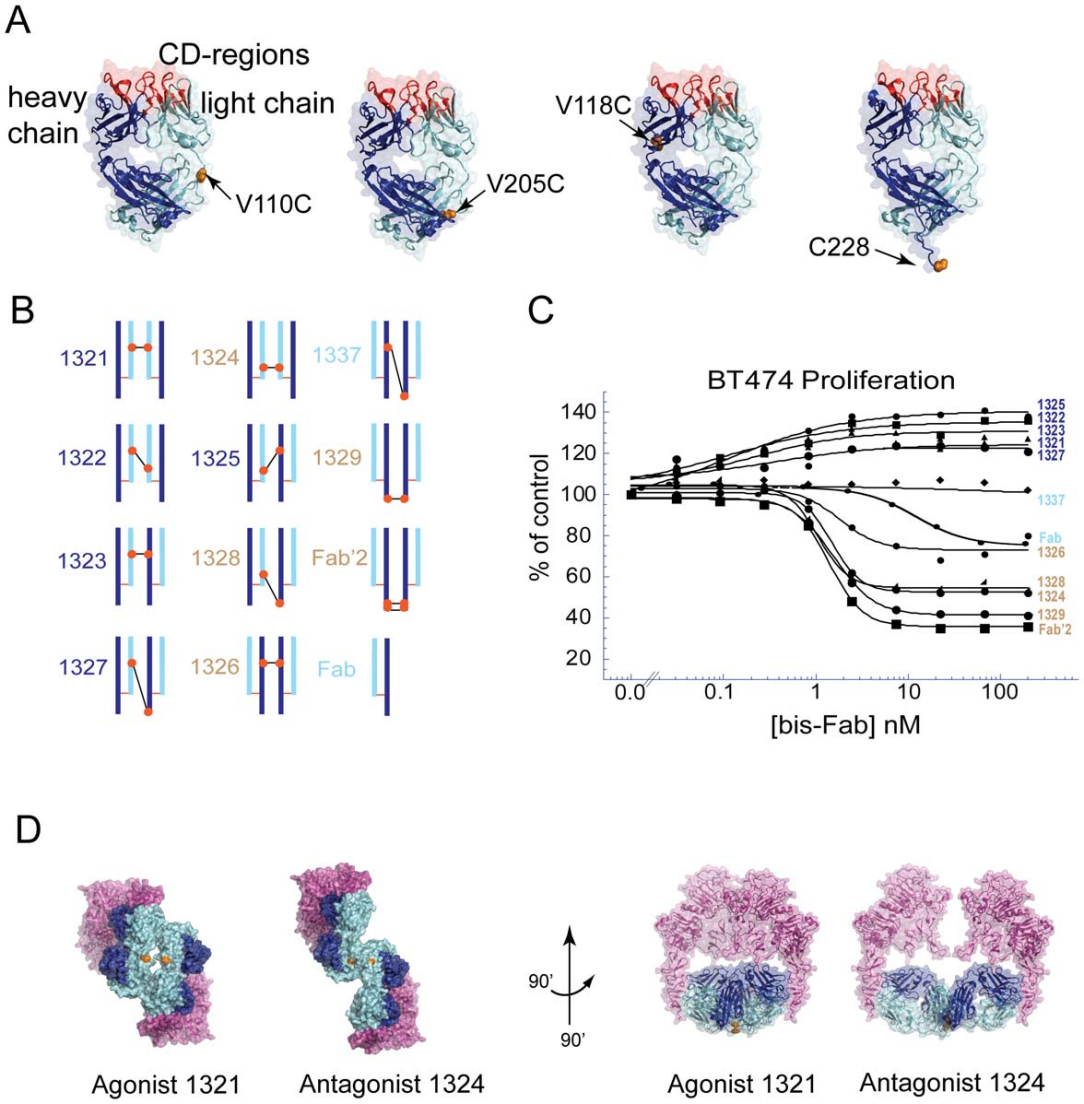
Trastuzumab bis-Fab structural analogs show a spectrum of cell-proliferation activities. (a) Four different thio-Fabs derived from trastuzumab were used to make ten bis-Fab analogs. Each thio-Fab mutant (LC-110, LC-205, HC-118, and HC-228) was reacted with the bis-maleimido crosslinker and recombined in a matrix format described in the Experimental Methods. Fabs were derived from several sources shown in **Figures S2A** and S**2B**. Each chain of the Fab is represented by a different color (dark blue - heavy chain and light blue - light chain) and the position of the cysteine used in coupling is denoted by the red dot. (b) The matrix-generated bis-Fab linkage analogs are shown diagrammatically in this Figure. Color coded numbers indicate the type of activity observed, where tan signifies antagonist, dark blue signifies agonist, and light blue signifies no activity. (c) BT474 cells were incubated with the indicated concentrations of bis-Fabs shown in (b). The degree of cell proliferation was assessed after 5 days using AlamarBlue staining. The results are reported as a percentage of maximum proliferation relative to untreated controls. The measured values for each test sample, as well as individual replicates, are shown as raw data in **Figure S3**. Color codes are the same as in (b). (d) A model of the complex formation between two HER2 extracelluar domains (ECD) and either an agonist (1321) or antagonist (1324) bis-Fab. Here is shown two light chain connected bis-Fabs with the heavy chain colored dark blue, the light chains colored lighter blue and the HER2 ECD colored magenta. In the left panel the two complexes are shown looking up at the membrane. The point of contact between the Fv-region of the Fab and the ECD is near the membrane. The HER2 protein terminates in this structure just prior to the point at which the transmembrane domain begins. The complex models are rotated 90 degrees on both the horizontal and vertical axis to produce this viewpoint. The plane of the membrane runs perpendicular to the page. The PBD ID Code used for model building was 1N8Z.

**Figure 3:**
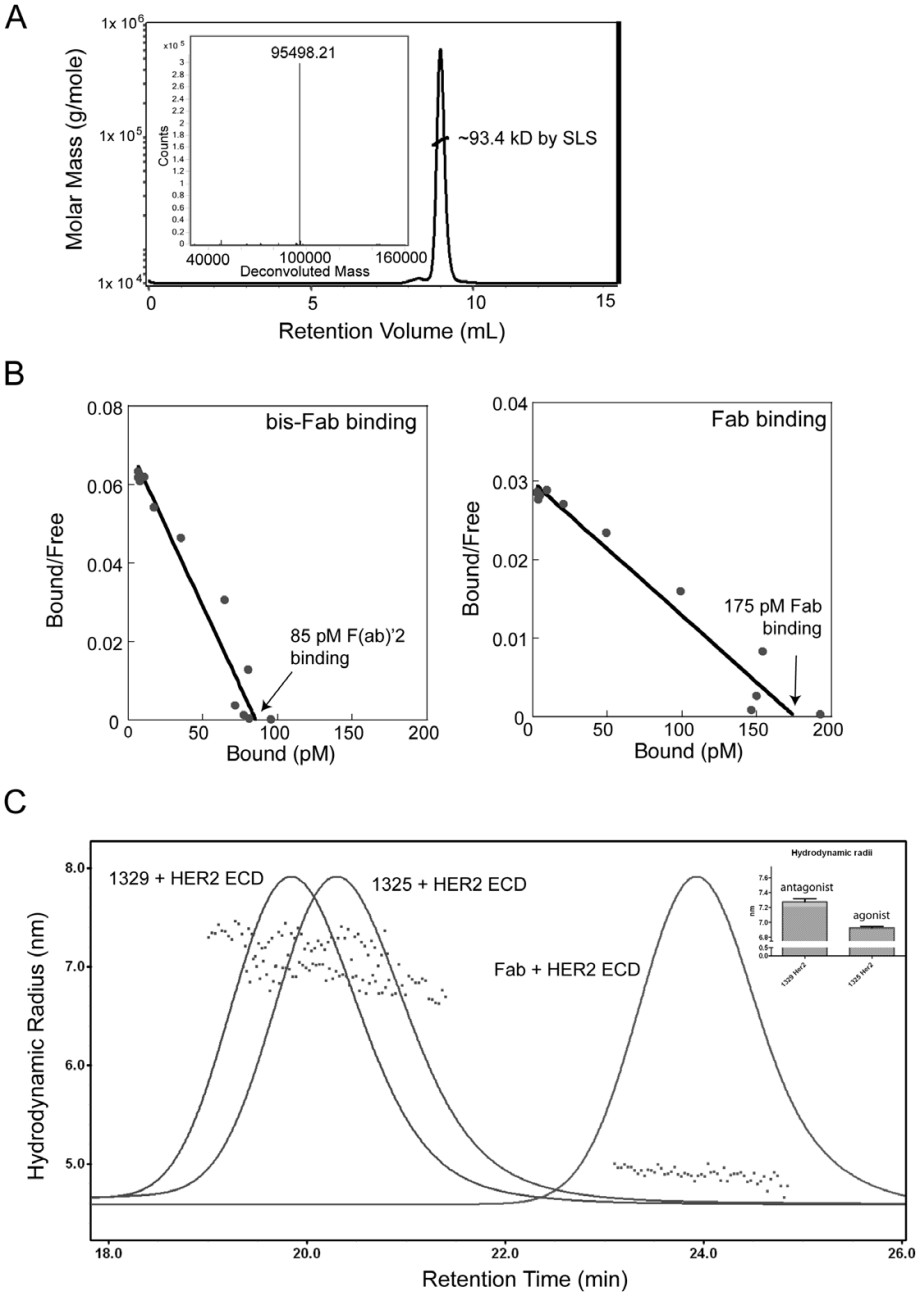
Quantitative analyses of bis-Fab biophysical and biochemical properties. (a) Bis-Fabs were analyzed for purity and aggregation using mass spectrometry and size exclusion chromatography (SEC). Shown here is a representative size exclusion chromatogram and mass spectrum for a bis-Fab, number 1325. The line across the major peak on the SEC indicates the molecular weight determined by static light scattering (SLS), around 93.4 kD. (b) To determine if bis-Fabs are binding with one Fab domain or two Fab domains to the cell surface, we performed Scatchard analysis using BT474 cells and radiolabeled bis-Fabs. All bis-Fabs showed cell surface affinities similar to that of the parent antibody trastuzumab, binding to two receptors per mole of bis-Fab. The data for number of receptor binding sites and affinity are shown here for both a representative bis-Fab and a single trastuzumab Fab domain. (c) Both an agonist and antagonist bis-Fab, 1329 and 1325, respectively, were bound to the receptor ECD in solution and analyzed by size exclusion chromatography coupled to a static light scattering detector. The complexes were analyzed by light scattering to determine the overall hydrodynamic radii, and an average of multiple measurements are shown in the histogram inset. The agonist complex had a radius slightly, but statistically significantly, smaller that the antagonist complex.

Because of the surprising activity of bis-Fab 1321, we questioned if this was the only form that behaved differently compared to the parent antibody. We generated trastuzumab Fabs with additional linkage attachment sites. We created a bis-Fab matrix that included trastuzumab thio-Fabs with cysteine mutations in the light chain at amino acids LC-110 and LC-205 and in the heavy chain at HC-118 and HC-228 (**Figure 2A**). These thio-Fabs were obtained through various production routes demonstrating the general accessibility of the technology (**Figure S2A and S2B**). The molecules were combined in a matrix format to produce ten unique structural analogs (**Figure 2B**). The variants exhibited a wide range of activities when tested in a BT474 cell-proliferation assay (**Figure 2C** **and Figure S3**). Several new agonistic molecules (EC_50_∼0.1 nM) were identified, including bis-Fab 1325 linked between positions LC-205 and HC-118, that showed enhanced cell proliferation activity compared to agonist bis-Fab 1321. Other bis-Fabs were either potent antagonists with activities comparable to trastuzumab (IC_50_∼1 nM) or produced little or no change in cell proliferation. To investigate the differences between molecules in more detail we first examined the quality of the proteins by standard techniques. Size exclusion chromatography coupled to a light scattering device indicated that the proteins were homogeneous preparations of approximately 95 kD monomeric proteins with no appreciable aggregation (**Figure 3A**). Mass spectrometric analysis also indicated that the proteins were of an exact molecular weight calculated from the theoretical combination of the two distinct thio-Fabs and the chemical crosslinker (**Figure 3A** **, inset**). Detailed Scatchard binding analyses showed that the agonistic bis-Fabs maintained the same affinity and number of binding sites as the parent F(ab′)_2_ (**Table 1**). The greater affinity relative to the monomeric Fab indicated that both arms of the bis-Fab, F(ab′)2 and trastuzumab bound two HER2 molecules at the same time. In addition, as shown in **Figure 3B** and **Table 1**, a significant difference between the number of binding sites per cell for Fabs (∼550,000) and bis-Fabs and trastuzumab (∼280,000) was measured. The two-fold difference reinforced that bis-Fabs and trastuzumab bound bivalently to the cells surface, forming a 2∶1 complex of receptor to bis-Fab. The fact that some bis-Fabs activated, and others inhibited, suggested that simple Fab-binding was not the key factor for inhibiting HER2, but rather the subtle nature of the complex formed.

To determine any solution state differences we carried out dynamic light scattering analysis of bis-Fabs alone or in complexes with the HER2 ECD. The agonistic bis-Fabs showed a trend toward being more compact molecules, as judged by their hydrodynamic radii (**Table 1**). Although the data for analysis of the bis-Fab-ECD complexes are statistically significant, reproducible and easily interpreted (**Figure 3C**), the differences are modest and do not offer a clear explanation for activity differences. The more striking structural difference among the antibody isomers, however, was the global orientation of the epitope-binding-Fv regions relative to one another. Because of the proximity of trastuzumab binding to the plasma membrane [20], the antibody binding may influence the transmembrane arrangement of HER2 dimers. To illustrate this in detail, we produced models of an agonist and antagonist in complex with the trastuzumab Fab based on the structure of the HER2 extracellular domain (ECD). For both the agonistic bis-Fab and the antagonistic bis-Fab we determined only one conformation in which both Fabs of each molecule bound to two receptors simultaneously (**Figure 2D**). The overall model indicated a more compact structure for the agonistic complex. The approximate radii based on the models confirmed a similar trend in size as determined by solution state analysis. Although these models represent only one possible configuration, we hypothesized that the orientation of HER2 molecules in the complex lead to the activation or inhibition of the intracellular kinase domain.

### Signaling pathway activation by trastuzumab-derived agonists

**Figure 4:**
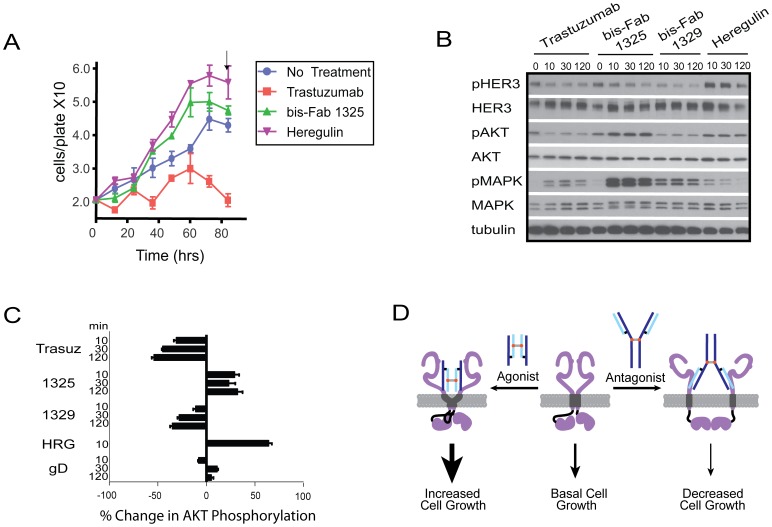
Analysis of bis-Fab agonist activity in BT474 cells. (a) A time course of cell growth activity in BT474 cells in the presence of 100 nM trastuzumab, 100 nM bis-Fab 1325, or 10 nM heregulin. BT474 were cultured in media containing 10% fetal bovine serum for up to 84 hours. At 12-hour intervals total number of cells were determined; three plates from each treatment group were counted for the total number of cells and plotted as the mean cell count. The error bars indicate standard deviation from the mean. At approximately 60 hours (indicated by the arrow) the cells reached confluence in the agonist treatment groups. (b) BT474 cells were treated with 100 nM of trastuzumab, 100 nM of bis-Fab 1325, or 100 nM of bis-Fab 1329 for 10, 30 and 120 minutes. At times indicated, cell lysates were prepared and analyzed by immunoblotting using phospho-specific antibodies for HER3, AKT, and MAPK as well as antibodies recognizing total protein. Data are representative of three independent experiments. (c) Quantification of AKT phosphorylation after treatment with 100 nM bis-Fab 1325, 100 nM trastuzumab, 2 nM heregulin and a non-specific control antibody (anti-gD) by PathScan p-AKT1 (S473) ELISA. All data points were collected in triplicates and the mean of the triplicate absorbance values were used to calculate the percent change in pAKT compared to untreated control group. The error bars indicate standard deviation from the mean. Data are representative of three independent experiments. (d) A model for the HER2 dimerization patterns induced by either the agonist antibody-analogs or trastuzumab. The diagram depicts three potential dimer conformations; 1) the basal state induced by high cell surface density, 2) the activated state induced by the bis-Fab agonist, and 3) the inhibited state stabilized by trastuzumab. The cell growth activity of agonist bis-Fabs may be due to stabilization of an allosterically activated conformation between HER2–HER2 dimers. Trastuzumab's antagonistic activity may arise from dimer orientations that favor the inactive allosteric interactions between kinases. A non-stabilized dimer may represent the basal state where interactions between the juxtamembrane (black line) loop of the activator kinase and the C-terminal lobe of the receiver kinase are not fully stabilized without agonist binding.

Upon receptor dimerization allosteric interactions regulate the kinase activity in HER-family members that in turn lead to the modulation of various downstream signaling pathways [23]. We investigated the influence of bis-Fabs on cell-signaling pathways compared to trastuzumab and a known agonist, heregulin. We evaluated the rate of cell growth of BT474 cells treated with the most active agonist bis-Fab 1325, trastuzumab or heregulin (**Figure 4A**). Heregulin is the ligand for HER3, which is the preferred heterodimerization partner for HER2. The growth stimulating activity of bis-Fab 1325 was comparable to heregulin whereby both agonists increased cell numbers at an accelerated rate compared to untreated cells. Within 36 hours, the bis-Fab 1325 and heregulin treated cells showed increased cell proliferation compared to control. The enhanced cell growth was sustained until confluency was reached for both agonist or heregulin treated cells (approximately 60 hours past treatment). The number of cells for bis-Fab 1325 treated cells increased by 243%, for heregulin-treated cells by 271% and for untreated controls by 176%, whereas trastuzumab treated cells, as expected, did not grow.

The increase in growth rate seen with bis-Fab 1325 coincided with activation of PI3K/AKT and MAPK signaling. As shown in **Figure 4B and 4C**, treatment with agonist bis-Fab 1325 for 10 minutes enhanced the phosphorylation of AKT and MAPK. In contrast, incubation with the antagonist bis-Fab 1329 decreased phosphorylation of AKT by about 30% similar to trastuzumab treatment (**Figure 4C**). Similar increases in AKT and MAPK phosphorylation were observed in other cells lines (SKBR3 and ZR75-30) as shown in **Figure S4**. Overall, the response of BT474 cells to bis-Fab 1325 was consistent with a HER2 pathway-activating molecule.

Despite HER2 activating properties, bis-Fab 1325 did not increase HER3 phosphorylation but rather induced a slow but noticeable decrease in levels of pHER3 (**Figure 4B**). This result was similar to trastuzumab treated cells but contrasted with heregulin activation, which increased phosphorylation of HER3 and indicated that bis-Fab 1325 stimulated downstream signaling pathways via HER2 in a HER3-independent manner. **Figure 4D** illustrates a possible mode of action for HER2 activation by trastuzumab analogs. In this model, both the agonist and antagonist antibodies bind HER2 with similar affinity and valency.

### HER2 phosphomapping and phospho-quantification

We examined whether the growth stimulatory activity of bis-Fab 1325 was, in fact, due to direct changes in the phosphorylation state of HER2. Because the basal phosphorylation level of HER2 amplified cells is high we used quantitative mass spectrometry as a more accurate measurement tool for receptor phosphorylation. This technique defined a complete signature of phosphorylation changes associated with agonistic activity at one time point. We first surveyed the basal phosphorylation state of HER2 in unstimulated BT474 cells by mass spectrometry. After immunoaffinity purification of HER2 from cell extracts and enzymatic digestion, peptides were identified by LC-MS/MS. Eighty-eight percent amino acid coverage of the intracellular domain of HER2 was obtained and seventeen phosphorylation sites in the basal state were identified. Phosphorylation was found to occur to varying levels on threonine, serine and tyrosine residues distributed throughout the entire intracellular domain. We confirmed phosphorylation on all five sites documented under the Uniprot entry (www.uniprot.org) for ERBB2_HUMAN and a comprehensive list of all sites identified are compared to annotation in other databases (**Table S1**).

Quantification of the level of phosphorylation in the basal state for fifteen sites was generated by mass spectrometry by including isotopically labeled synthetic peptides in LC-MS/MS analyses of tryptic digests of isolated HER2 [24]. The amount of basal phosphorylation ranged from less than 1% at sites in the kinase domain to greater than 90% at Ser-1054 in the C-terminal tail (**Table 2** and **Table S3**). This survey allowed a snapshot of the early phosphorylation changes induced by either agonist or antagonist treatment in BT474 cells.

**Table 2:** Quantification of HER2 phosphorylation and the differences after treatment.

Site*	% Phosph.†	Difference From Basal‡		
		Trastuzumab	Bis-Fab 1325	Heregulin
T701	48.2	−0.4	18.1	20.3
S728	0.6	0.1	0	0
Y735	0.1	0	0	0
Y877	0.1	0	0	0
Y1005	29.6	−6.8	15.3	18.8
S1054	87.7	1.1	3.7	4.2
S1073	29.3	−5.8	17.1	15.1
S1078	35.9	6.8	9.6	10.4
S1083	37.1	9.3	6.3	7.5
S1100	1.3	−0.3	0.1	0.4
T1103	1.3	0.5	0.1	0.4
Y1139	84.1	2.8	10.3	6.7
Y1151	6.8	3.9	5.4	−1.3
T1240	40.6	−15.1	−22.3	−9.8
Y1248	17.8	21.1	14.9	−4

* Phosphorylation sites were identified by HER2 phosphomapping.

† Quantitative mass spectrometry (MS) was done to determine the basal level of phosphorylation at each site. The absolute amount of phosphorylated peptide was compared to the total of phosphorylated and non-phosphorylated peptides to calculate the percent phosphorylation (% Phosph.)

‡ Ten minutes after treatment with the indicated molecule, quantitative MS was done to determine the % phosphorylation. This was subtracted from the % phosphorylation in the basal state to provide the mean difference after treatment reported here. This time point was chosen because maximal phosphorylation of AKT was shown to occur within ten minutes after agonist treatment (**Figure 4**).

**Figure 5:**
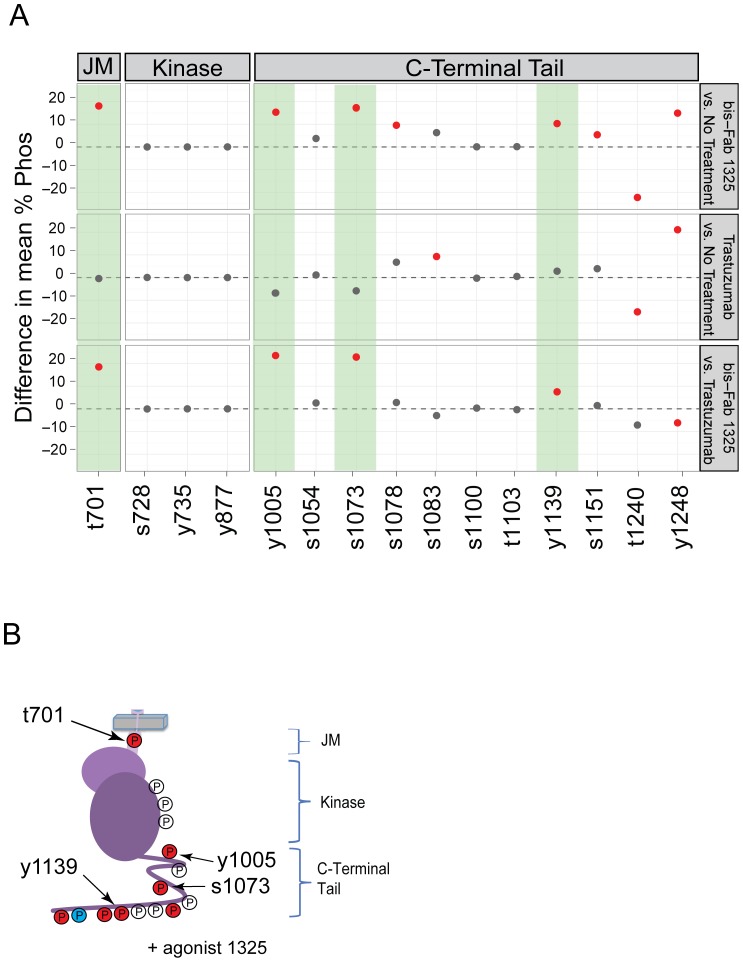
Changes in HER2 phosphorylation are associated with agonist activity. (a) This panel compares phosphorylation differences at fifteen sites in HER2 after treatment with either the agonist bis-Fab 1325 or trastuzumab. The top panel shows differences between agonist treatment and no treatment (basal phosphorylation). Seven of the sites showed statistically significant increases in phosphorylation (red dots above the line), five sites showed no change, and one site decreased. The same comparison is made between trastuzumab (antagonist) and no treatment shown in the second panel. A third comparison is made between bis-Fab 1325 and trastuzumab. Here, sites that show statistically significant differences between the treatments are indicated by red dots. Those phosphorylation sites that are significantly higher than trastuzumab treatment are highlighted with green background. The results were analyzed with a Tukey-Kramer all-pairwise test and the error bars indicate the 95% confidence level. Methods used are described in detail in the Supplementary Information. **Figure S5** shows the raw data consisting of nine mass spec measurements for each treatment and phosphorylation site. Red color is used to indicate a statistically significant difference between the two samples with a P-value of <0.05. (b) This illustration of the HER2 intracellular domains shows phosphorylation sites identified by mass spectrometry. Phosphorylation sites colored orange denote sites with measureable increases, blue for decreases and uncolored for no change after agonist bis-Fab 1325 treatment. Phosphorylation sites that are significantly higher in agonist treatments compared to trastuzumab treatments are indicated by arrows.

Interestingly, the level of phosphorylation of many sites did not change significantly upon trastuzumab treatment (**Table 2** and **Figure S5**). In contrast, more changes were evident with agonist treatments, for example, phosphorylation at sites T701, Y1005, S1073, and Y1139 increased above basal levels by 50 to 100%. (**Figure 5A** and **Table 2**) Two of these sites clearly correlated with agonist vs. antagonist treatment (Y1005 and S1073). A Tukey-Kramer pairwise comparison between treatment and no treatment demonstrated that bis-Fab 1325 and trastuzumab produced distinctly different phosphorylation patterns (**Figure 5B**): phosphorylation of T1240 decreased whereas Y1248 increased in response to both trastuzumab and bis-Fab 1325.

## Discussion

One important contribution provided by these bis-Fab molecules is a specific understanding that changes in the orientation of the Fv-regions relative to one another in an antibody (or antibody-like) molecule can result in profoundly different biological activities. Recently, several reports have described disulfide-connectivity variants in antibodies found both in nature and during synthetic development [25]. These variants were suspected of having different biological functions due to their altered domain structures. Using our technology to systematically alter the arrangement of one Fab arm relative to the other arm, we have been able to show that a robust structure-activity relationship exists between different antibody-isomers.

Because HER2 is an important therapeutic target, how the receptor activates cell growth pathways has been extensively studied. The mechanism of trastuzumab inhibition has also been rigorously investigated. Despite these efforts, questions still remain about how the receptor is activated and the mechanism of inhibition imparted by the antibody. Our structure-function analyses and biochemical studies of bis-Fab isomers that have the same affinity but induce opposite activities suggested that alternative active conformations can be achieved by simply altering the geometry and relative orientation of two HER2 proteins (**Figures 2D** and **4D**). Thus, one consequence of the bivalent binding of trastuzumab to HER2 might be to fix the orientation of the kinases in an inhibited state. This is supported by our antibody binding analysis (Scatchard analysis) comparing trastuzumab, bis-Fab 1329, bis-Fab 1325, and the trastuzumab Fab and F(ab′)_2_ binding [26]. All bivalent trastuzumab-related molecules bind to HER2 with the same cell surface affinity (K_d_∼1.5 nM) which is greater than the affinity of the monovalent Fab (K_d_∼6 nM). In addition, the Fab domain alone has very little biological activity (**Figure 2C**). This is consistent with recent crystallographic studies showing the occurrence of both inactive and active conformations of HER-family kinase dimers [23], [27], [28], [29]. A similar model has been proposed for an antibody that stimulates receptor phosphorylation through bivalent binding to a different epitope [30], [31]. Using bis-Fab 1325 to directly activate HER2, we have established that both the MAPK pathway and AKT pathways are activated and only certain phosphorylation sites on the HER2 intracellular domain increase in their level of phosphorylation after activation. Because no other chemical probe exists to directly activate native HER2, a thorough analysis of the signaling events following agonist treatment could be important. In support of this, a recent report expressing recombinant HER2-FKBP fusions in MCF10A cells using AP1510 to induce dimerization of FKPB alters sensitivity to trastuzumab [32]. Such analyses provide new considerations for breast cancer combination therapy in HER2 positive patients, especially those where evidence of HER2-homodimers have been observed.

The finding that a simple linkage analog of an antibody can have a dramatic influence on function is a new concept. This highlights the structural and functional plasticity of a molecule that possesses two protein-protein interaction domains. During antibody discovery, the engineering of target affinity into antibody CDRs is often a primary consideration. The effects of the orientation of Fab domains on target complexes are not often integrated into antibody design. We have defined a process by which antibody activities can be altered without modifying the complementarity regions and target affinity. This may explain in some cases of antibody humanization why antibodies may lose their agonistic or antagonistic function but not their capacity to effectively bind their target [33]. In addition, changes in the hinge region have been previously noted to influence activity by altering domain flexibility. These examples illustrate that domain flexibility and positioning are important aspects of the functional properties of an antibody. To this end, we have begun using structural models of Fabs and their respective targets to identify sets of cysteine positions for different Fv-to-Fv distances and geometry in antibodies to enhance their functional properties.

From our study we suggest that disulfide-isoforms that impact Fab-domain flexibility and geometry have critical importance both in nature and in antibody development. Our approach offers a simple way to modulate global structure with several important advantages to other antibody design techniques such as diabodies that use peptide-based linkers. Rational design can be imparted by using Fab structural models and linkers of defined length. By combining rational design with simple combinatorial synthesis, this technique offers access to a greater repertoire of isomeric variants for creating molecules with unique functions. The linker described herein is amenable to additional modifications with functional groups such as PEG, cytotoxic drugs, and fluorescent probes to expand its useful application. Exploiting the multidomain feature of antibodies through linkage engineering can expand the diversity of tools for better understanding biological process and provide potential therapeutics with unique activity.

## Supporting Information

Figure S1 **A process for the synthesis of bis-Fabs.** Using Fabs containing a single unpaired cysteine (thio-Fabs), we developed a site-specific crosslinking strategy to make bispecific molecules composed of two such thio-Fabs. The unpaired cysteine was incorporated into either the heavy or light chain of the Fab outside of the combining region by site-directed mutagenesis. Alternatively, the cysteines of the hinge region of the heavy chain can be used as a crosslinking site by producing a F(ab′)_2_ from the intact antibody by pepsin digestion followed by mild reduction. Thio-Fabs were prepared for crosslinking by removing any existing thio-adducts or dimers by reduction and reoxidation. The reduced and oxidized thio-Fab was then reacted with an excess of the bis-maleimido crosslinker and the reaction mixture was subsequently isolated by gel filtration. The monomeric species containing the thio-Fab-crosslinker complex was reacted with the second thio-Fab at a concentration greater than 5 mg/mL. The reaction was monitored by mass spectrometry and the final product (bis-Fab) composed of the crosslinked Fabs was isolated by a second gel filtration. The earlier eluting peak on the second gel filtration contained the ∼100 kDa bis-Fab irreversibly crosslinked through the two unpaired cysteines. Product purity was determined by mass spectrometry and SDS-PAGE under reducing and non-reducing conditions.
(TIF)

Figure S2 **A matrix for combinatorial synthesis of trastuzumab bis-Fab variants.** (a) Trastuzumab thio-Fabs were derived from three different sources: 1) light chain 110-Cys was obtained by expression in *E. coli*, 2) light chain 205-Cys and heavy chain 118-Cys were expressed as full length thio-mAbs in mammalian cells containing the cysteine mutation and subsequently obtained by digestion with lysine-endopeptidase and isolation by anion exchange chromatography, and 3) heavy chain hinge-Cys (228/230-Cys) was obtained from the native trastuzumab molecule by pepsin digestion C-terminal to the hinge-disulfide bonds followed by reduction and controlled chemistry using a thiol-reactive reagent N-ethylmaleimide (NEM) to produce a thio-Fab at the hinge region containing a single thiol. (b) Trastuzumab was digested with pepsin to release the F(ab′)_2_, which was subsequently purified by anion exchange chromatography. F(ab′)_2_ from trastuzumab was reduced with tris(2-carboxyethyl)phosphate (TCEP) to liberate the two Fab domains followed by oxidation with dehydroascorbic acid in the presence of 5 mM EDTA at pH 5.8. The reduced and oxidized thio-Fabs were reacted with 1 equivalent of NEM (N-ethylmaleimide) as shown in the second panel. This reaction produced three products, a thio-Fab with two NEM irreversible adducts, a species with one NEM adduct and a single thiol, and an unreacted species with two thiols. The right panel shows mass spectrometric analysis of the reaction products indicating the presence of the three species. The MW of NEM is 125 Da and the unreacted thio-Fab is 48651 Da. The reaction mixture produced after addition of NEM was subsequently reacted with an excess of the crosslinker bis-Mal (XL) shown in the third panel. As determined by mass spectrometry, two new species of thio-Fab products were identified, one containing one bis-Mal crosslinker and one NEM (MW 49323) and the second containing only one bis-Mal crosslinker (+XL). The bis-Mal crosslinker reacting with the trio-Fab containing two thiols resulted in the formation of a cyclic-complex containing one bis-Mal crosslinker that had efficiently reacted with both cysteines. This species was no longer thio-active and possessed no reactive maleimide group. Only the product containing one crosslinker and one NEM reacted with a second thio-Fab in the second coupling step of the trio-Fab assembly process described in Figure S1.
(TIF)

Figure S3 **Raw data for cell proliferation in response to trastuzumab bis-Fabs.** Here we show the raw data that is represented in Figure 2C. The data are shown directly as relative fluorescence units (RFU) measured in the assay. Because several assay plates are needed to test all the samples, assay results from each plate are shown in separate plots. This allows viewing of the raw data without normalization.
(TIF)

Figure S4 **Analysis of bis-Fab agonist activity in ZR75-30 and SKBR3 cells.** ZR75-30 and SKBR3 cells were treated with 100 nM of trastuzumab, 100 nM of bis-Fab 1325, or 100 nM of bis-Fab 1329 for 10, 30 and 120 minutes. At times indicated, cell lysates were prepared and analyzed by immunoblotting using phospho-specific antibodies for HER3, AKT, and MAPK as well as antibodies recognizing total protein. Data are representative of three independent experiments.
(TIF)

Figure S5 **Scatter plots showing the total level of phosphorylation of each phosphosite.** These plots show levels of phosphorylation at each site in each treatment group. Each of three gel bands from the immunoisolations was derived from independent biological replicates. The gel bands containing HER2 were analyzed by mass spectrometry in triplicate. Thus, for each treatment group there are nine data points, three mass spec replicates of three biological replicates. Each phosphorylation percentage is shown as a scatter plot and the mean is indicated with a bar. Four treatment groups, No Treatment (NoTx), Trastuzumab, bis-Fab 1325 (1325), and heregulin (Hrg) are shown together for each phosphosite. The data are shown left to right in the order they occur in the sequence of HER2.
(TIF)

Table S1 **Coverage of HER2 phosphorylation sites.**
(DOC)

Table S2 **A list of the synthetic peptides used for HER2 quantification.** Isotopic label mass addition was based on the residue of incorporation (orange). Phosphorylated residues are shown in blue. A mixture of 50 fmol/µL of these 19 peptides was used for HER2 phosphoryl-quantification upon treatment.
(DOC)

TableS3 **Quantification of HER2 phosphorylation. Blue letters indicate phosphorylated residues.**
(DOC)
