# Supplementary figures and images for: Reorienting the Fab Domains of Trastuzumab Results in Potent HER2 Activators
Source: PLoS One. 2012 Dec 20;7(12):e51817. doi: 10.1371/journal.pone.0051817 (PMC3527469; doi:10.1371/journal.pone.0051817)

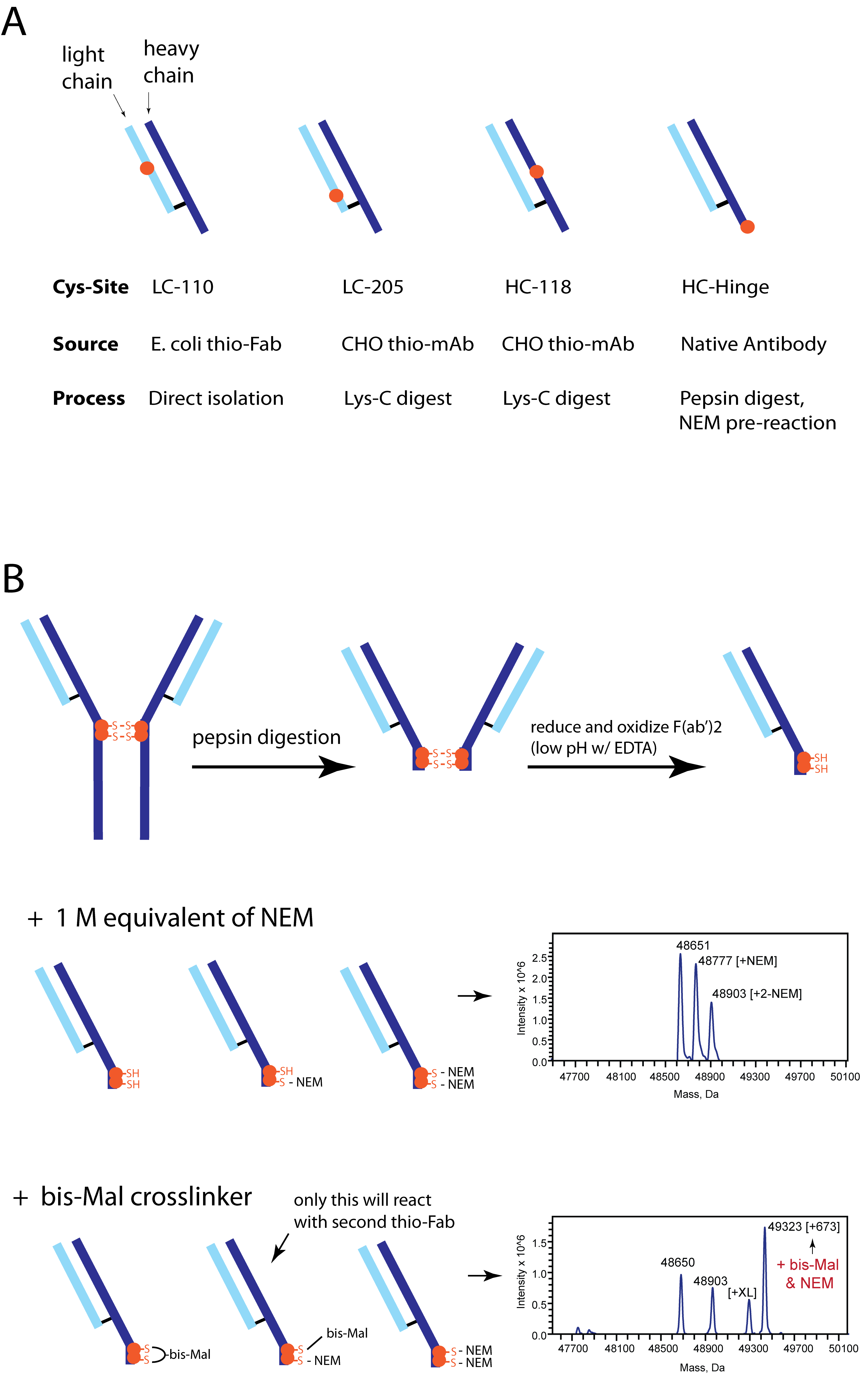

Supplement: Figure S2 — A matrix for combinatorial synthesis of trastuzumab bis-Fab variants. (a) Trastuzumab thio-Fabs were derived from three different sources: 1) light chain 110-Cys was obtained by expression in E. coli, 2) light chain 205-Cys and heavy chain 118-Cys were expressed as full length thio-mAbs in mammalian cells containing the cysteine mutation and subsequently obtained by digestion with lysine-endopeptidase and isolation by anion exchange chromatography, and 3) heavy chain hinge-Cys (228/230-Cys) was obtained from the native trastuzumab molecule by pepsin digestion C-terminal to the hinge-disulfide bonds followed by reduction and controlled chemistry using a thiol-reactive reagent N-ethylmaleimide (NEM) to produce a thio-Fab at the hinge region containing a single thiol. (b) Trastuzumab was digested with pepsin to release the F(ab′)2, which was subsequently purified by anion exchange chromatography. F(ab′)2 from trastuzumab was reduced with tris(2-carboxyethyl)phosphate (TCEP) to liberate the two Fab domains followed by oxidation with dehydroascorbic acid in the presence of 5 mM EDTA at pH 5.8. The reduced and oxidized thio-Fabs were reacted with 1 equivalent of NEM (N-ethylmaleimide) as shown in the second panel. This reaction produced three products, a thio-Fab with two NEM irreversible adducts, a species with one NEM adduct and a single thiol, and an unreacted species with two thiols. The right panel shows mass spectrometric analysis of the reaction products indicating the presence of the three species. The MW of NEM is 125 Da and the unreacted thio-Fab is 48651 Da. The reaction mixture produced after addition of NEM was subsequently reacted with an excess of the crosslinker bis-Mal (XL) shown in the third panel. As determined by mass spectrometry, two new species of thio-Fab products were identified, one containing one bis-Mal crosslinker and one NEM (MW 49323) and the second containing only one bis-Mal crosslinker (+XL). The bis-Mal crosslinker reacting with the trio [file pone.0051817.s002.tif]

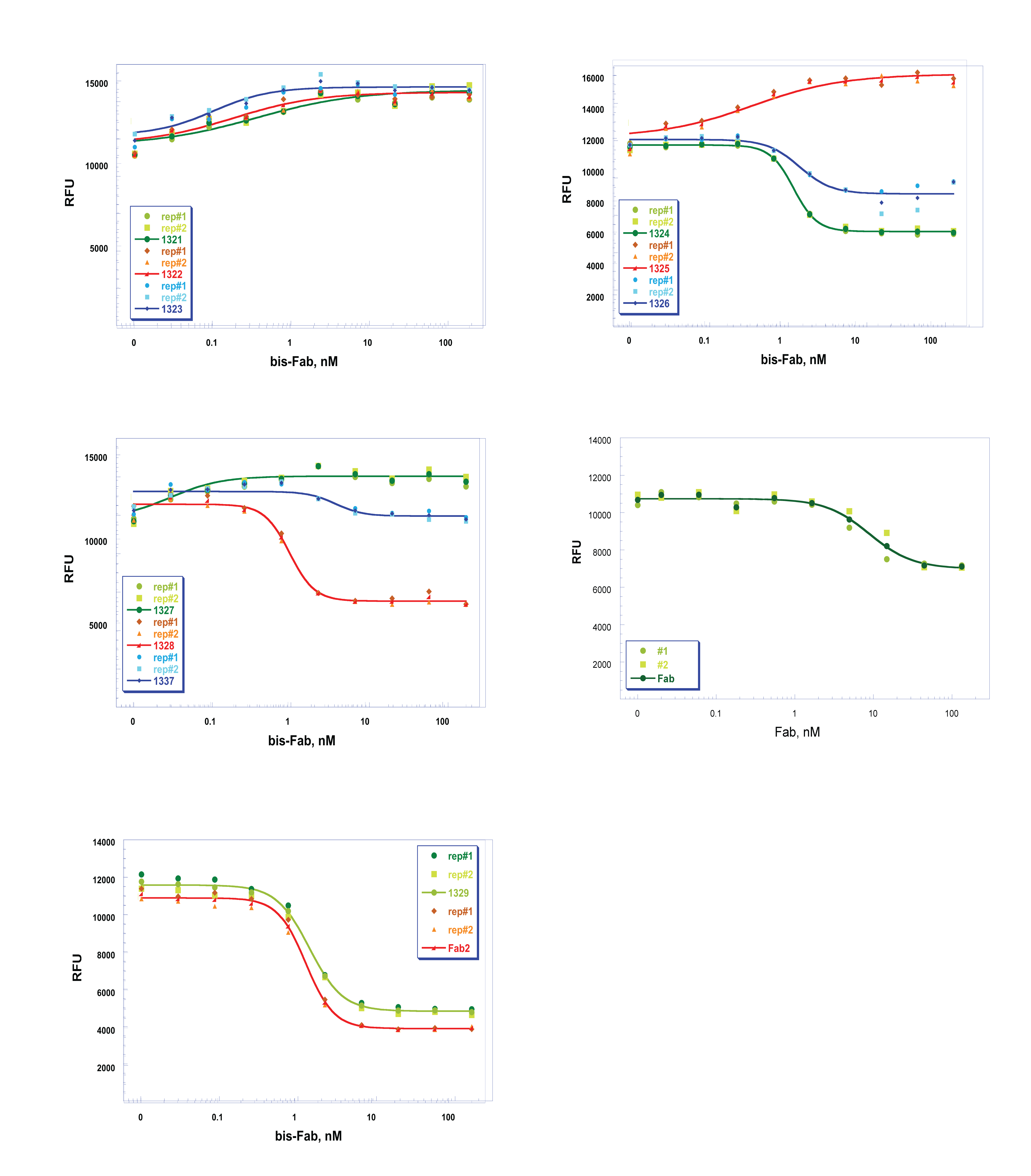

Supplement: Figure S3 — Raw data for cell proliferation in response to trastuzumab bis-Fabs. Here we show the raw data that is represented in Figure 2C. The data are shown directly as relative fluorescence units (RFU) measured in the assay. Because several assay plates are needed to test all the samples, assay results from each plate are shown in separate plots. This allows viewing of the raw data without normalization. (TIF) [file pone.0051817.s003.tif]

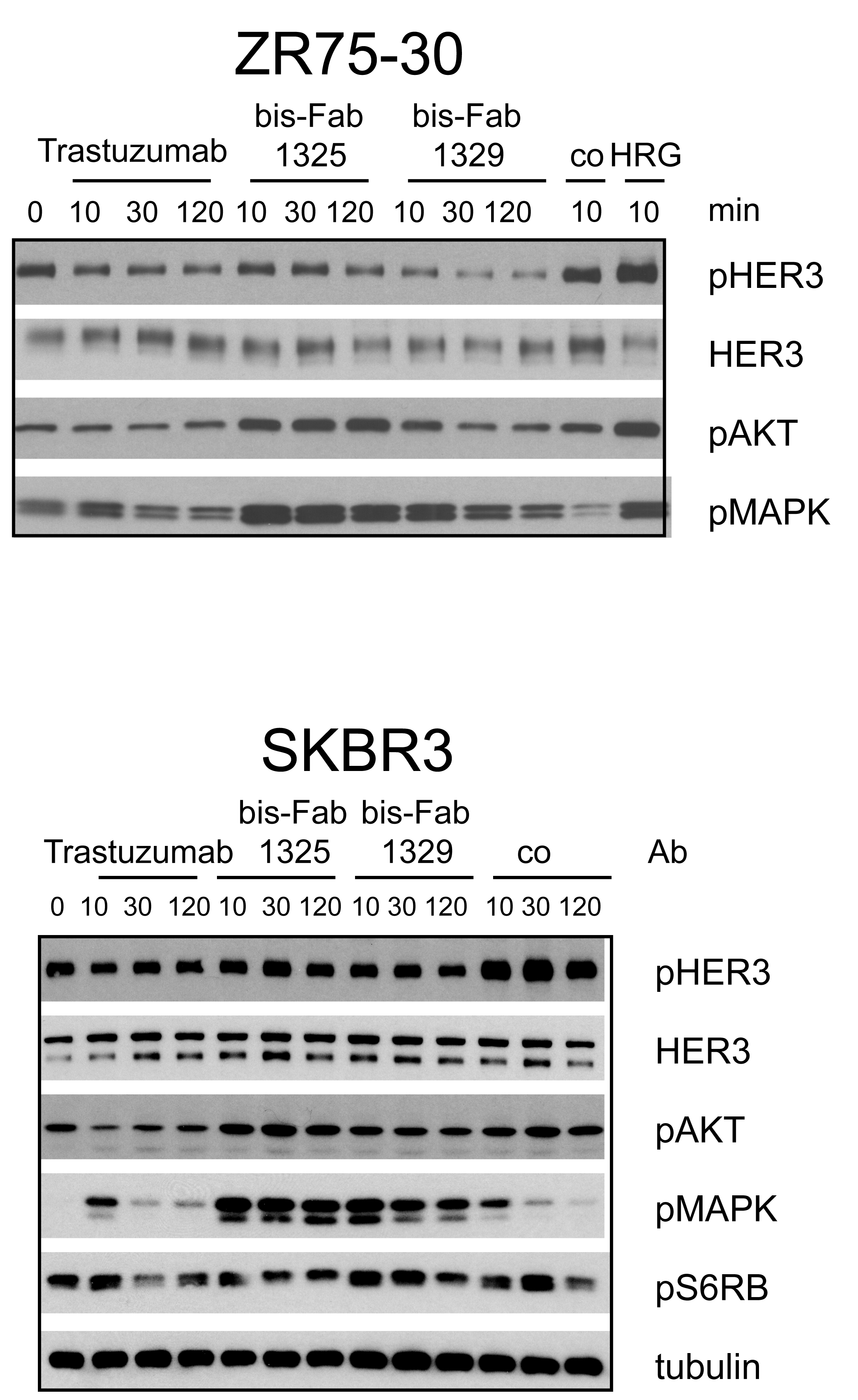

Supplement: Figure S4 — Analysis of bis-Fab agonist activity in ZR75-30 and SKBR3 cells. ZR75-30 and SKBR3 cells were treated with 100 nM of trastuzumab, 100 nM of bis-Fab 1325, or 100 nM of bis-Fab 1329 for 10, 30 and 120 minutes. At times indicated, cell lysates were prepared and analyzed by immunoblotting using phospho-specific antibodies for HER3, AKT, and MAPK as well as antibodies recognizing total protein. Data are representative of three independent experiments. (TIF) [file pone.0051817.s004.tif]

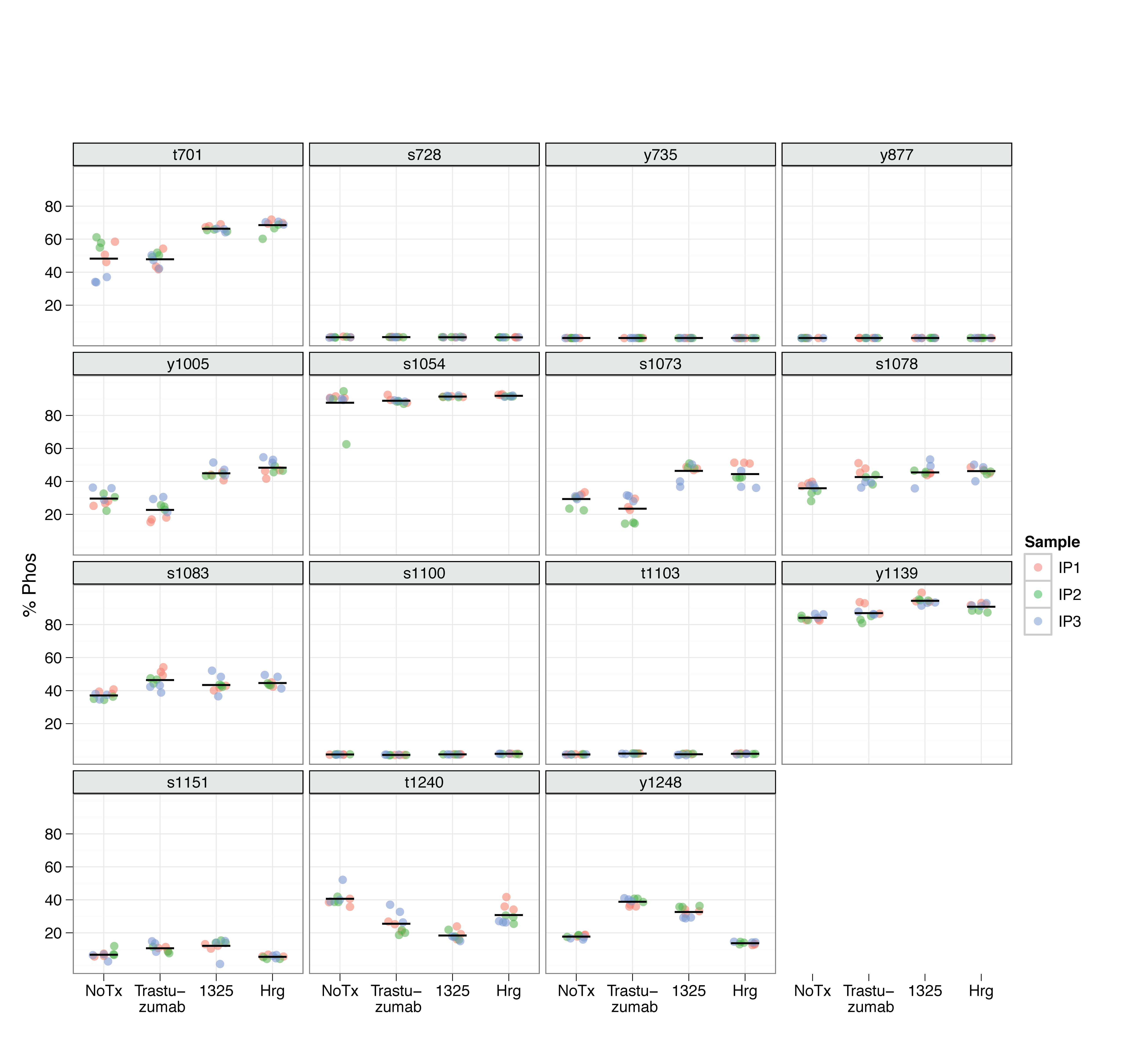

Supplement: Figure S5 — Scatter plots showing the total level of phosphorylation of each phosphosite. These plots show levels of phosphorylation at each site in each treatment group. Each of three gel bands from the immunoisolations was derived from independent biological replicates. The gel bands containing HER2 were analyzed by mass spectrometry in triplicate. Thus, for each treatment group there are nine data points, three mass spec replicates of three biological replicates. Each phosphorylation percentage is shown as a scatter plot and the mean is indicated with a bar. Four treatment groups, No Treatment (NoTx), Trastuzumab, bis-Fab 1325 (1325), and heregulin (Hrg) are shown together for each phosphosite. The data are shown left to right in the order they occur in the sequence of HER2. (TIF) [file pone.0051817.s005.tif]
