# Supplementary material for: Reorienting the Fab Domains of Trastuzumab Results in Potent HER2 Activators
Source: PLoS One. 2012 Dec 20;7(12):e51817. doi: 10.1371/journal.pone.0051817 (PMC3527469; doi:10.1371/journal.pone.0051817)
Supplement: Table S1 — Coverage of HER2 phosphorylation sites. (DOC) [file pone.0051817.s006.doc]

**Supplemental Table 1.** Coverage of HER2 phosphorylation sites.

| **Site** | **Current Analysis** | **Expasy** | **phosphosite.org** | **phosida.org** |
| --- | --- | --- | --- | --- |
| 686 | Not Covered |  | X | X |
| 701 | X |  |  |  |
| 728 | X |  | X |  |
| 735 | X | X | X | X |
| 877 | X |  | X | X |
| 998 | (X)* |  | X |  |
| 1003 | (X)* |  |  |  |
| 1005 | X |  | X |  |
| 1023 | (X)† |  | X |  |
| 1054 | X | X | X | X |
| 1066 | X |  |  |  |
| 1073 | X |  |  |  |
| 1078 | X |  |  |  |
| 1083 | X |  |  |  |
| 1100 | X |  |  |  |
| 1103 | X |  | X | X |
| 1112 | Covered ‡ |  | X | X |
| 1127 | Covered ‡ |  | X |  |
| 1139 | X | X | X |  |
| 1151 | X |  | X |  |
| 1166 | X |  | X |  |
| 1172 | Not Covered |  | X |  |
| 1196 | Covered ‡ | X | X | X |
| 1221/1222 | Covered ‡ |  | X | X |
| 1240/1242 | X |  |  |  |
| 1248 | X | X | X | X |

* s998 & t1003 MS-MS containing spectrum were found that localized phosphorylation to these sites on the same peptide as y1005. Changes at these sites were not quantifiable due to co-elution.

† y1023 was identified in peptide 1007-1046 in some but not all samples indicating phosphorylation. Peptide was seen as +4 or +5 ion and eluted late or not at all.

‡ y1112 and y1127 were covered in peptide 1112-1153. On this peptide, two phosphorylation sites were identified as y1139 and s1151. A second, minor peak was observed by full MS for the doubly phosphorylated peptide, however, no corresponding tandem MS was acquired to localize these sites. y1196 and y1221/y1222 were seen in the large tryptic peptide covering residues 1183-1230 in some runs, but no phosphorylation was found to be present.
