# Supplementary material for: Reorienting the Fab Domains of Trastuzumab Results in Potent HER2 Activators
Source: PLoS One. 2012 Dec 20;7(12):e51817. doi: 10.1371/journal.pone.0051817 (PMC3527469; doi:10.1371/journal.pone.0051817)
Supplement: Table S2 — A list of the synthetic peptides used for HER2 quantification. Isotopic label mass addition was based on the residue of incorporation (orange). Phosphorylated residues are shown in blue. A mixture of 50 fmol/µL of these 19 peptides was used for HER2 phosphoryl-quantification upon treatment. (DOC) [file pone.0051817.s007.doc]

**Supplemental Table 2.** A list of the synthetic peptides used for HER2 quantification. Isotopic label mass addition was based on the residue of incorporation (orange). Phosphorylated residues are shown in blue. A mixture of 50 fmol/µL of these 19 peptides was used for HER2 phosphoryl-quantification upon treatment.

|  |  | **add** | HER2 Synthetic Peptide | **Heavy monoisotopic** |
| --- | --- | --- | --- | --- |
| **+13C** | **+15N** | **mass** |  | **MH+** |
| 5 | 1 | 6.0138 | VLGSGAFGT***V***YK | 1204.6605 |
| 5 | 1 | 6.0138 | VLGSGAFGT***Vy***K | 1284.6268 |
|  |  |  |  |  |
| 4 | 2 | 6.0075 | LLDIDETEYHAD***GG***K | 1681.7885 |
| 4 | 2 | 6.0075 | LLDIDETE***y***HAD***GG***K | 1761.7548 |
|  |  |  |  |  |
| 5 | 1 | 6.0138 | EGPLPAARPAGATLER***P***K | 1837.0323 |
| 5 | 1 | 6.0138 | EGPLPAARPAGA***t***LER***P***K | 1916.9986 |
|  |  |  |  |  |
| 5 | 1 | 6.0138 | SGGGDLTLGLEPSEEEA***P***R | 1919.9225 |
| 5 | 1 | 6.0138 | ***s***GGGDLTLGLEPSEEEA***P***R | 1999.8888 |
|  |  |  |  |  |
| 6 | 1 | 7.0171 | GLQSLPTHDPSP***L***QR | 1652.8827 |
| 6 | 1 | 7.0171 | GLQ***s***LPTHDPSP***L***QR | 1732.8491 |
|  |  |  |  |  |
| 5 | 1 | 6.0138 | GTPTAENPEYLGLDV***P***V | 1777.8887 |
| 5 | 1 | 6.0138 | GTPTAENPE***y***LGLDV***P***V | 1857.8550 |
|  |  |  |  |  |
| 5 | 1 | 6.0138 | LLQETELVEPLTPSGAM***P***NQAQMR | 2659.3462 |
| 5 | 1 | 6.0138 | LLQETELVEPL***t***PSGAM***P***NQAQMR | 2739.3125 |
|  |  |  |  |  |
| 6 | 1 | 7.0171 | FVVIQNEDLGPASP***L***DSTFYR | 2375.1991 |
| 6 | 1 | 7.0171 | FVVIQNEDLGPASP***L***DSTF***y***R | 2455.1654 |
|  |  |  |  |  |
| 6 | 1 | 7.0171 | SPLAPSEGAGSDVFDGD***L***GMGAAK | 2256.0562 |
| 6 | 1 | 7.0171 | SPLAP***s***EGAGSDVFDGD***L***GMGAAK | 2336.0225 |
| 6 | 1 | 7.0171 | SPLAP***s***EGAG***s***DVFDGD***L***GMGAAK | 2415.9888 |
