# Supplementary material for: Reorienting the Fab Domains of Trastuzumab Results in Potent HER2 Activators
Source: PLoS One. 2012 Dec 20;7(12):e51817. doi: 10.1371/journal.pone.0051817 (PMC3527469; doi:10.1371/journal.pone.0051817)
Supplement: TableS3 — Quantification of HER2 phosphorylation. Blue letters indicate phosphorylated residues. (DOC) [file pone.0051817.s008.doc]

**Supplemental Table 3.** Quantification of HER2 phosphorylation. Blue letters indicate phosphorylated residues.

| Site | Pep-tide | Peptide Sequence | Quantita-tion | Basal | Her-ceptin | Bis-Fab | Hereg-ulin |
| --- | --- | --- | --- | --- | --- | --- | --- |
|  |  |  |  | % Phos  ± SD | %  Phos  ± SD | % Phos  ± SD | %  Phos  ± SD |
|  |  |  |  |  |  |  |  |
| *t701* | 690-713 | LLQETELVEPL***t***PSGAMPNQAQMR | AQUA | 48.2 ± 10.9 | 47.8 ± 4.5 | 66.3 ± 1.5 | 68.5 ± 3.4 |
| *s728* | 725-736 | VLG***s***GAFGTVYK | AQUA* | 0.59 ± 0.20 | 0.65 ± 0.08 | 0.59 ± 0.11 | 0.54 ± 0.05 |
| *y735* | 725-736 | VLGSGAFGTV***y***K | AQUA | 0.09 ± 0.02 | 0.09 ± 0.01 | 0.10 ± 0.01 | 0.08 ± 0.01 |
| *y877* | 869-883 | LLDIDETE***y***HADGGK | AQUA | 0.1 ± 0.03 | 0.12 ± 0.01 | 0.12 ± 0.06 | 0.11 ± 0.26 |
| *y1005* | 986-1006 | FVVIQNEDLGPASPLDSTF***y***R | AQUA | 29.6 ± 4.7 | 22.7 ± 5.3 | 44.9 ± 3.0 | 48.3 ± 4.1 |
| *s1054* | 1054-1072 | ***s***GGGDLTLGLEPSEEEAPR | AQUA | 90.8 ± 1.7 | 88.8 ±1.5 | 91.4 ± 0.4 | 91.9 ± 0.6 |
| *s1054, s1066* | 1054-1072 | ***s***GGGDLTLGLEP***s***EEEAPR | none | NA | NA | NA | NA |
| *s1073* | 1073-1096 | ***s***PLAPSEGAGSDVFDGDLGMGAAK | AQUA* | 16.9 ± 2.1 | 8.8 ± 1.7 | 31.6 ± 6.1 | 27.0 ± 5.3 |
| *s1078* | 1073-1096 | SPLAP***s***EGAGSDVFDGDLGMGAAK | AQUA | 0.82 ± 0.15 | 1.01 ± 0.42 | 0.84 ± 0.10 | 1.05 ±0.23 |
| *s1083* | 1073-1096 | SPLAPSEGAG***s***DVFDGDLGMGAAK | AQUA* | 5.9 ± 0.64 | 8.6 ± 1.1 | 5.3 ± 0.5 | 5.4 ± 1.0 |
| *s1073, s1078* | 1073-1096 | ***s***PLAP***s***EGAGSDVFDGDLGMGAAK | AQUA* | 3.9 ± 1.1 | 3.6 ± 0.7 | 6.2 ± 1.2 | 5.8 ± 0.9 |
| *s1078, s1083* | 1073-1096 | SPLAP***s***EGAG***s***DVFDGDLGMGAAK | AQUA | 22.7 ± 2.2 | 30.5 ± 3.5 | 28.0 ± 3.3 | 26.3 ± 2.7 |
| *s1073, s1078, s1083* | 1073-1096 | ***s***PLAP***s***EGAG***s***DVFDGDLGMGAAK | AQUA* | 9.6 ± 2.8 | 8.5 ± 5.9 | 10.5 ± 2.1 | 13.2 ± 1.9 |
| *s1100* | 1097-1111 | GLQ***s***LPTHDPSPLQR | AQUA | 1.30 ± 0.12 | 1.04 ± 0.12 | 1.36 ± 0.04 | 1.69 ± 0.14 |
| *t1103* | 1097-1111 | GLQSLP***t***HDPSPLQR | AQUA* | 1.32 ± 0.12 | 1.83 ± 0.14 | 1.46 ± 0.37 | 1.69 ± 0.22 |
| *y1139* | 1112-1153 | YSEDPTVPLPSETDGYVAPLTCSPQPE***y***VNQPDVRPQPPSPR | Label free | 76.8 ± 3.4 | 75.5 ±3.9 | 82.4 ±3.9 | 85.5 ±2.4 |
| *s1139, s1151* | 1112-1153 | YSEDPTVPLPSETDGYVAPLTCSPQPE***y***VNQPDVRPQPP***s***PR | Label free | 6.8 ± 2.4 | 10.7 ± 2.5 | 12.1 ± 4.4 | 4.9 ± 1.9 |
| *t1166* | 1154-1171 | EGPLPAARPAGA***t***LERPK | none | NA | NA | NA | NA |
| *t1240/ 42* | 1239-1255 | G[tPt]AENPEYLGLDVPV | AQUA* | 40.6 ± 4.6 | 25.5 ± 6.1 | 18.3 ± 2.9 | 30.7 ± 5.5 |
| *y1248* | 1239-1255 | GTPTAENPE***y***LGLDVPV | AQUA | 17.8 ± 1.0 | 38.8 ± 2.0 | 32.7 ± 3.0 | 13.8 ± 0.7 |

**** synthetic peptide used has another phosphorylation site on the same peptide***
